# Dapagliflozin attenuates hypoxia/reoxygenation-caused cardiac dysfunction and oxidative damage through modulation of AMPK

**DOI:** 10.1186/s13578-021-00547-y

**Published:** 2021-02-26

**Authors:** Kun-Ling Tsai, Pei-Ling Hsieh, Wan-Ching Chou, Hui-Ching Cheng, Yu-Ting Huang, Shih-Hung Chan

**Affiliations:** 1 Department of Physical Therapy, College of Medicine, National Cheng Kung University, Tainan, Taiwan; 2 Institute of Allied Health Sciences, College of Medicine, National Cheng Kung University, Tainan, Taiwan; 3 Department of Anatomy, School of Medicine, China Medical University, Taichung, Taiwan; 4 Department of Internal Medicine, College of Medicine, National Cheng Kung University Hospital, National Cheng Kung University, Tainan, Taiwan

**Keywords:** Dapagliflozin, Ischemia/reperfusion injury, AMPK

## Abstract

**Background:**

Emerging evidence demonstrated dapagliflozin (DAPA), a sodium-glucose cotransporter 2 inhibitor, prevented various cardiovascular events. However, the detailed mechanisms underlying its cardioprotective properties remained largely unknown.

**Results:**

In the present study, we sought to investigate the effects of DAPA on the cardiac ischemia/reperfusion (I/R) injury. Results from in vitro experiments showed that DAPA induced the phosphorylation of AMPK, resulting in the downregulation of PKC in the cardiac myoblast H9c2 cells following hypoxia/reoxygenation (H/R) condition. We demonstrated that DAPA treatment diminished the H/R-elicited oxidative stress via the AMPK/ PKC/ NADPH oxidase pathway. In addition, DAPA prevented the H/R-induced abnormality of PGC-1α expression, mitochondrial membrane potential, and mitochondrial DNA copy number through AMPK/ PKC/ NADPH oxidase signaling. Besides, DAPA reversed the H/R-induced apoptosis. Furthermore, we demonstrated that DAPA improved the I/R-induced cardiac dysfunction by echocardiography and abrogated the I/R-elicited apoptosis in the myocardium of rats. Also, the administration of DAPA mitigated the production of myocardial infarction markers.

**Conclusions:**

In conclusion, our data suggested that DAPA treatment holds the potential to ameliorate the I/R-elicited oxidative stress and the following cardiac apoptosis via modulation of AMPK, which attenuates the cardiac dysfunction caused by I/R injury.

## Introduction

In spite of the advances made in diagnostic and therapeutic approaches, the death rates and complications attributable to cardiovascular diseases (CVD) remained disturbingly high [1]. It has been shown that the long-term prognosis of patients with acute myocardial infarction (AMI) was not benign, especially when their left ventricular ejection fraction was ≤ 45% [2]. Hence, a vast amount of research effort has been devoted to the studies on myocardium salvage and reduction of infarction size. To date, the standard treatments of AMI are reperfusion strategies using either thrombolytic therapy or primary percutaneous coronary intervention for maintaining the patency of the infarct-related coronary artery [3]. However, these approaches may inevitably result in undesirable ischemia/reperfusion (I/R) injury.

During the ischemia phase, the metabolism of the affected cells was altered and their energy-dependent function was impaired due to the reduced adenosine triphosphate (ATP) availability, which eventually leading to calcium overload, structural disorganization, and cell death. Later, the restoration of blood flow further exacerbated tissue damage as the unremitting generation of reactive oxygen species (ROS) aggravated cell death [4]. Mitochondrial ROS production has been shown to regulate redox signal and sensed by various enzymes, and one of them was adenosine 5’-monophosphate activated protein kinase (AMPK) [5]. It has been demonstrated that AMPK activation triggered a peroxisome proliferator-activated receptor gamma coactivator 1-alpha (PGC-1α)-dependent antioxidant response and the interplay between AMPK and PGC-1α participated in the control of ROS homeostasis [5]. Besides, results from studies using transgenic mice with impaired cardiac AMPK activation [6] or direct activation of AMPK by a small molecule [7] both supported that AMPK possessed the properties to regulate myocardial metabolism, and limit the apoptosis and cardiac dysfunction following I/R injury.

Dapagliflozin (DAPA) is a selective sodium-glucose cotransporter 2 (SGLT2) inhibitor, which can be used to reduce plasma glucose as it blocks glucose resorption in the kidney and induces glycosuria for patients with type 2 diabetes mellitus (DM) [8]. Recently, robust data from multiple clinical trials revealed the cardioprotective effects of DAPA. For instance, DAPA has been shown to be implicated in the improvement of myocardial function and reduced all-cause mortality in DM patients with chronic heart failure [9, 10]. Moreover, DAPA treatment led to a lower rate of cardiovascular death or hospitalization for DM patients who were at risk for atherosclerotic CVD [11] or had previous myocardial infarction [12]. Nevertheless, the molecular mechanism underlying its function in the heart has not been elucidated. Given that SGLT2 was mainly expressed in the kidney [13] and absent in the heart [14], it seemed SGLT2 may be excluded as a potential explanation for the direct cardioprotective effects of DAPA. In fact, one of the recent studies has revealed that DAPA induced anti-inflammatory responses in cardiofibroblasts stimulated with lipopolysaccharides through AMPK activation without the involvement of SGLT2 [14]. As such, we sought to examine whether DAPA exerted its beneficial effects on the heart subjected to I/R injury via AMPK and investigated the associated mechanisms.

To this end, we utilized cardiac myoblast H9c2 cells and assessed the AMPK phosphorylation in response to hypoxia/reoxygenation (H/R) condition. Subsequently, we dissected its signaling pathway and demonstrated how DAPA limited ROS production and apoptosis. Additionally, we verified that DAPA mitigated the cardiac damage caused by I/R injury and preserved the cardiac function in vivo.

## Materials and methods

### Cell culture and reagents

H9c2 cells, which are myoblasts cells from rat myocardium, were purchased from the American Type Culture Collection. H9c2 cells were cultured with Dulbecco’s modified Eagle’s medium (DMEM) supplemented with 10% fetal bovine serum (FBS) and penicillin (50 IU/mL)/streptomycin (50 µg/mL). A 0.25% (w/v) Trypsin-0.53 mM ethylenediaminetetraacetic acid (EDTA) solution was used to passage cells. Cells were cultured in humidified air with 5% CO^2^ at 37°C. FBS and EDTA were purchased from Gibco (NY, USA). The 5,58,6,68-tetraethylbenzimidazolcarbocyanine iodide (JC-1), Ro-32-0432, diphenyleneiodonium chloride (DPI), anti-Rac1 antibody (05-389; 1:1000) and anti-phospho-AMPK antibody (SAB4503754; 1:1000), were purchased from Sigma (MO, USA). Dihydroethidium (DHE) was obtained from Thermo Scientific (MA, USA). Anti-β-actin (sc-47,778; 1:50,000), anti-AMPK (sc-74,461; 1:1000), anti-Nox-2 (sc-74,514; 1:1000), anti-phospho‐PKC (sc-377,565; 1:1000), anti-PKC (sc-8393; 1:1000), anti-phospho-p53 (sc-51,690; 1:1000), anti-Bax (sc-20,067; 1:2000), anti-Bcl2 (sc-27,382; 1:1000), anti-cytochrome c (sc-13,156; 1:1000), anti-Na/K ATPase (sc-48,345; 1:10,000), and anti‐PGC-1α (sc-518,025; 1:1000) were obtained from Santa Cruz Biotechnology ( CA, USA). Horseradish peroxidase (HRP)-conjugated anti‐rabbit and anti‐mouse secondary antibodies were purchased from Transduction Laboratories (CA, USA). Rat Lactate Dehydrogenase (LDH) ELISA Kit (MBS269777) was bought from MyBioSource (BC, Canada). Rat Creatine Kinase MB isoenzyme (CK-MB) ELISA Kit (CSB-E14403r) was bought from CUSABIO (TX, USA).

### Primary culture of cardiomyocytes

 Newborn rats were sacrificed by cervical dislocation within 24 h. Their ventricles were washed with PBS, minced into small pieces and digested with 0.25% pancreatin and 0.1% collagenase in humidified air with 5% CO2 at 37 °C for 15 mins. Lysates were obtained via centrifugation at 1200 rpm for 10 mins. The pellet was resuspended in DMEM/F-12 with 10% FBS and penicillin.

### Hypoxia and ischemia induction

H9c2 cells were washed two times with PBS to remove glucose and serum in the culture medium. In control cells, the medium was replaced with glucose-free DMEM; in DAPA-treated cells, the medium was replaced with DAPA containing glucose-free DMEM. The cells were transferred to a hypoxia chamber containing of 95% N2 and 5% CO^2^ for 1 h. After hypoxia exposure, the cells were placed in a 5% CO^2^ and 95% O^2^ incubator for reoxygenation with high-glucose DMEM containing of 10% FBS; in DAPA treated cells, DAPA was added to high-glucose DMEM. In animal study, a total of 24 healthy, 200–250 g weight, 8–9 weeks aged male Sprague-Dawley (SD) rats were bought from BioLASCO Taiwan. Animals were housed in the temperature-controlled room (21–22 °C) and fed with regular food in the animal center at National Cheng Kung University. SD rats were randomly numbered and assigned into 3 groups. The vehicle or DAPA (0.1 mg/kg per day) was administered by the oral route. DAPA was dissolved in 60% propylene glycol and was given daily by gavage for 5 days before and 4 days after operation till sacrifice. Our animal study was performed at the Laboratory Animal Center, College of Medicine, National Cheng Kung University. This animal study was approved by the Institutional Animal Care and Use Committee (IACUC) of National Cheng Kung University (IACUC No. 109,067). For ischemia induction in animals, the rats were anesthetized intramuscularly using the mixture of 10:1 tiletamine/zolazepam (Zoletil, Virbac, France) and xylazine (Rompun, Bayer, USA). The dosage of anesthesia is 0.1 ml Zoletil/100 g body wight. Next, the heart was accessed by left thoracotomy, and the pericardium was removed. Ischemia was induced via the ligation of the left anterior descending coronary artery (LAD) with a 6−0 silk suture. After 1 h, ligation of LAD was released to allow reperfusion. The animals in the control group underwent the same surgical procedures but without LAD ligation. Four days after surgery, the animals were sacrificed for further experiments. The rats were sacrificed by injecting overdose of Zoletil mixed Rompun intramuscularly and then inhaling overdose of isoflurane.

###  Protein isolation, tissue extraction and Western blotting assay

Protein expression levels were investigated by Western blotting. Total protein was isolated from cells of the left ventricle. After sacrifice, the hearts of all the animals were collected. The tissue of left ventricle was washed 2 times with PBS buffer, and then 100 mg of tissue was cut for homogenization with radioimmunoprecipitation assay lysis buffer. The homogenates were centrifuged at 13,000×*g* for 30 mins, and the supernatant was collected and placed at − 80 °C until use. The proteins were transferred to a polyvinylidene difluoride membrane after the proteins were separated by electrophoresis on sodium dodecyl sulfate polyacrylamide gels. The membranes were stained with ponceau S to confirm the quality of protein transferring and the concentration of loading proteins. After then, the membranes were blocked by buffer at 37 °C for 1 h. Then, the membranes were incubated with primary antibodies at 4 °C for 18 h followed by hybridization with HRP-conjugated secondary antibodies for 1 h. The intensities were quantified by densitometric analysis. Plasma was obtained through blood collection for lactate dehydrogenase (LDH) and creatine kinase-MB (CK-MB) assay.

### Determination of cardiac functional parameters

Twenty-four animals were used in this study, with eight animals in each group. Four days after operation, echocardiography was performed to assess cardiac function. Anesthesia was induced by placing animals in a box with 5% isoflurane for 5 mins and then maintained in 1.0–1.5% isoflurane through a facemask. Isoflurane-anesthetized animals were placed in a supine position. Echocardiographic data were collected by a Vevo 770 microimaging system with a 25-MHz probe (VisualSonics, Toronto, ON, Canada). Parameters were collected based on the M-mode and two-dimensional images obtained in the parasternal long and short axis views at the level of the papillary muscles.

### Apoptotic assay

Apoptotic cells were analyzed by the ApopTag® Peroxidase In Situ Apoptosis Detection Kit (Calbiochem). After H/R treatment, cells were rinsed twice in PBS before fixation with 4% paraformaldehyde at room temperature for 30 mins. Next, cells were washed in PBS before incubation in the prepared solution (0.1% Triton X-100, 0.1% sodium citrate) for 5 mins. Cells were then incubated with terminal deoxynucleotidyl transferase dUTP nick end labeling (TUNEL) reaction mixtures in a humidified atmosphere for 1 h at 37 °C in the dark, washed in PBS, and analyzed by flow cytometry. The BioVision CaspGLOW™ Fluorescein Active Caspase-3 Staining Kit (Milpitas, CA, USA) was used for detection of active caspase 3. For investigating apoptosis in animal cardiac tissues, tissues were soaked in 4% paraformaldehyde. Paraffin-embedded heart was cut into 2-µm-thick sections. TUNEL staining was performed for apoptosis. In brief, the tissue sections were deparaffinized in xylene, rehydrated through a graded alcohol series (100%, 90%, 85%, and 75%), and then rinsed in PBS (pH 7.2). A DNA fragmentation detection kit (FragEL; Calbiochem) was used to detect apoptotic cells in cardiac tissue sections using TUNEL assay. The numbers of positive cells were measured and averaged from three different fields of view with the heaviest TUNEL staining in the infarction zone.

### Investigation of mitochondrial membrane potential

The JC-1 is widely used to study mitochondrial membrane potential. In healthy cells, JC-1 concentrates in the mitochondrial matrix where it forms red fluorescent aggregates. In apoptotic and necrotic cells, JC-1 exists in monomeric form and stains cells green. After stimulation of H/R, cells were rinsed with DMEM and then loaded with JC-1 (5 µM). After 30 mins incubation at room temperature, cells were assayed by flow cytometry.

### Mitochondrial biogenesis

Real-time polymerase chain reaction (PCR) assay was performed to investigate mitochondrial deoxyribonucleic acid (mtDNA) content. The primers for mitochondrial complex II were sense primer 5’-CAAACCTACGCCAAAATCCA-3’ and antisense primer 5’-GAAATGAATGAGCCTACAGA-3’. The primers for β-actin were sense primer 5’-AGGTCATCACTATTGGCAACGA-3’ and antisense primer 5’-CACTTCATGATGGAATTGAATGTAGTT-3’. The result of real-time PCR was assayed by SYBR Green on an ABI 7000 sequence detection system according to the protocol.

### NADPH oxidase activity assay

The lucigenin method was used to determine the nicotinamide adenine dinucleotide phosphate hydrogen (NADPH) oxidase activity in cells. The crude membrane fraction of cells was prepared by the Mem-PER™ Plus Membrane Protein Extraction Kit Thermo Scientific (MA, USA). In brief, cells were collected by centrifugation with 1300 rpm for 10 mins. Then, 0.75 mL of permeabilization buffer was added into the cell pellet and incubated tubes for 10 mins at 4 °C with constant mixing. The supernatant containing cytosolic proteins were collected by centrifugation with 12,000 rpm for 15 mins. The cytosolic proteins were transferred to a new tube. Next, 0.5 mL of solubilization buffer was added to the pellet and incubated tubes for 30 mins at 4 °C with constant mixing. The supernatant containing solubilized membrane proteins were collected by centrifugation with 12,000 rpm for 15 mins. The total protein concentration was adjusted to 1 mg/mL. An aliquot of 200 µL protein was incubated in the presence of 5 µM lucigenin and 100 µM NADPH. The luminescence was measured after 10 mins incubation by a plate reader (VICTOR3; PerkinElmer) to determine the relative changes in NADPH oxidase activity.

### Measurement of ROS formation

ROS generation in H9c2 cells was tested using DHE. Cells were pre-treated with or without DAPA for 2 h. Next, cells were exposed to H/R. After removing the medium from the wells, cells were incubated with 10 µM DHE for 30 mins. The fluorescence intensity was calculated by a flow cytometry.

### Transfection with small interfering RNA

AMPK siRNA, PGC-1-α siRNA (AMPK ON-TARGET plus SMART pool and PGC-1-α ON-TARGET plus SMART pool) and negative control siRNA (ON‐TARGET plus non‐targeting pool) were purchased from Dharmacon. Two days after transfection, cells were treated with a reagent as indicated for further experiments.

### Statistical analyses

Results are expressed as the mean ± standard deviation (SD). Statistical analyses were performed using analysis of variance, followed by Turkey’s test. A p value < 0.05 was considered statistically significant.

## Results

### DAPA stimulates AMPK phosphorylation in H9c2 cells under normoxia and hypoxia/reoxygenation condition

**Fig. 1:**
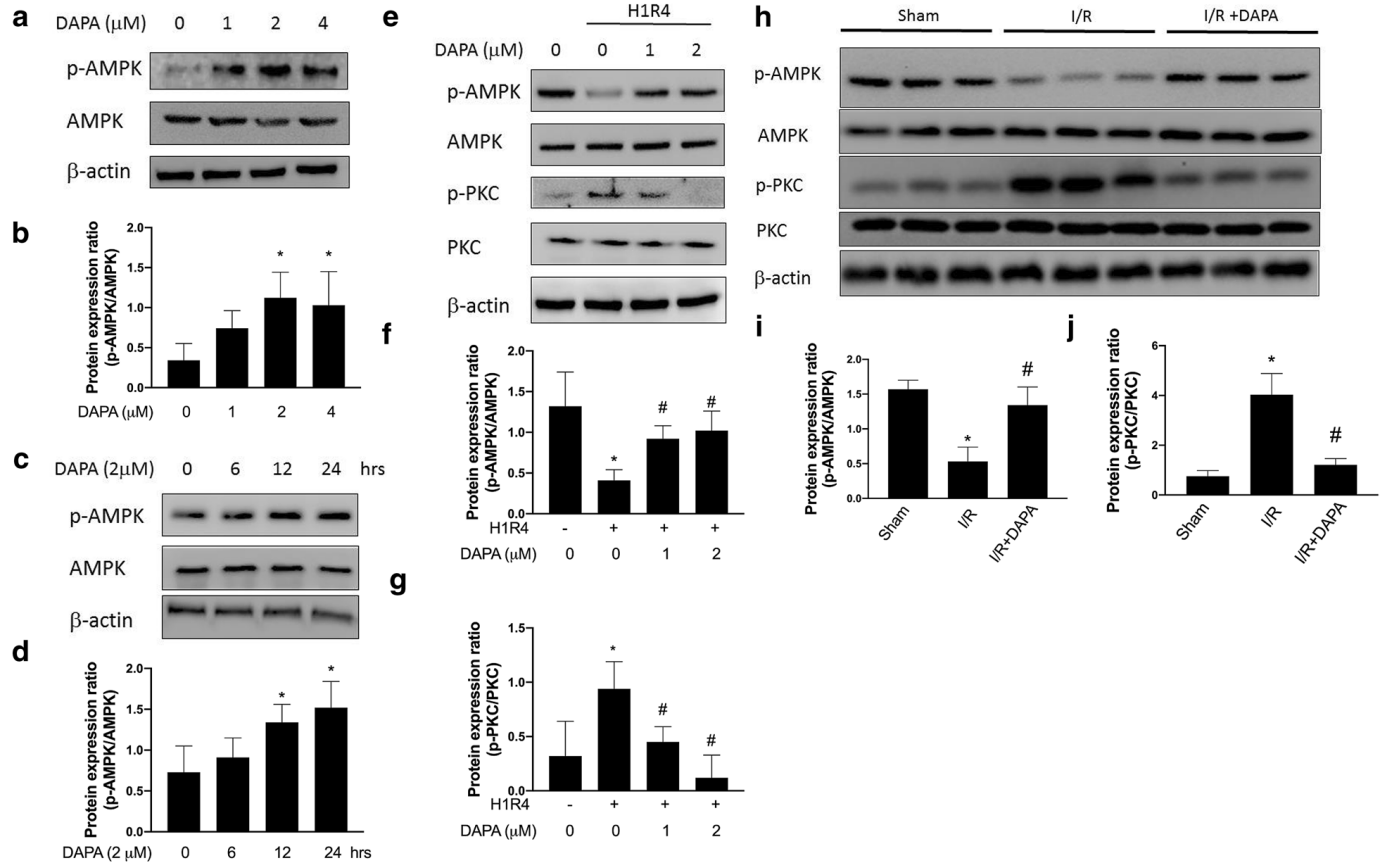
Administration of dapagliflozin (DAPA) increases the phosphorylation of AMPK and suppresses the phosphorylation of PKC under hypoxia/reoxygenation condition. The expression levels of phosphorylated AMPK in H9c2 cells treated with DAPA were enhanced in dose-dependent (**a,** **b**) and time-dependent (**c**, **d**) manners. Representative Western blot images (**e**) and relative densitometric bar graphs of phosphorylated-AMPK/AMPK (**f**) and phosphorylated-PKC/PKC (**g**) in H9c2 cells exposed to hypoxia for 1 h and reoxygenation for 4 h (H1R4) were shown. The data were presented as the mean ± SD of three biological replicates at three separate times. Representative Western blot image (**h**) and protein expression levels of phosphorylated-AMPK, AMPK, phosphorylated-PKC, and PKC in ventricular tissue from sham control, ischemia/reperfusion (I/R), and I/R plus DAPA treatment animals, eight animals in each group, were shown (**i**, **j**). (* indicating *p* < 0.05 compared with the control group; # indicating *p* < 0.05 compared to H1R4 condition or I/R without DAPA treatment)

 The expression of phosphorylated AMPK elevated dose-dependently in cardiac myoblast H9c2 cells following DAPA treatment under normoxia condition (Fig. 1a, b). Likewise, the treatment of H9c2 cells with DAPA resulted in a time-dependent increase in the expression of phosphorylated AMPK (Fig. 1c, d). We showed that the activation of AMPK was decreased in response to 1 h hypoxia with subsequent 4 h reoxygenation (H1R4) (Fig. 1e, f), which was consistent with a previous study showing a decline in the level of phosphorylated AMPK was observed after 24 h of reoxygenation [15]. It has been shown that activation of AMPK was associated with the reduced phosphorylation of protein kinase C (PKC) [16], which was in conformity with our data (Fig. 1e, g). With the administration of DAPA, the expression of phosphorylated AMPK increased after H1R4, while the phosphorylation of PKC was suppressed in a dose-dependent manner (Fig. 1e, g). These results suggested that DAPA treatment was able to restore the insufficient AMPK and repress the PKC activation in cardiac myoblast H9c2 cells following H/R condition. In order to prove this hypothesis, we conducted ischemia/reperfusion (I/R) injury model in animals which were subjected to 1 h of LAD occlusion followed by reperfusion for 4 days with or without DAPA treatment. The phosphorylated AMPK levels were decreased in ventricular tissues with I/R, however, DAPA treatment reversed I/R-repressed phosphorylated AMPK levels (Fig. 1h, i). In addition, the phosphorylated PKC levels were up-regulated in ventricular tissues with I/R, as expected, DAPA treatment mitigated I/R-elevated phosphorylated PKC levels (Fig. 1h, j).

### DAPA prevents the hypoxia/reoxygenation-induced oxidative stress through AMPK/ PKC/ NADPH oxidase pathway

**Fig. 2:**
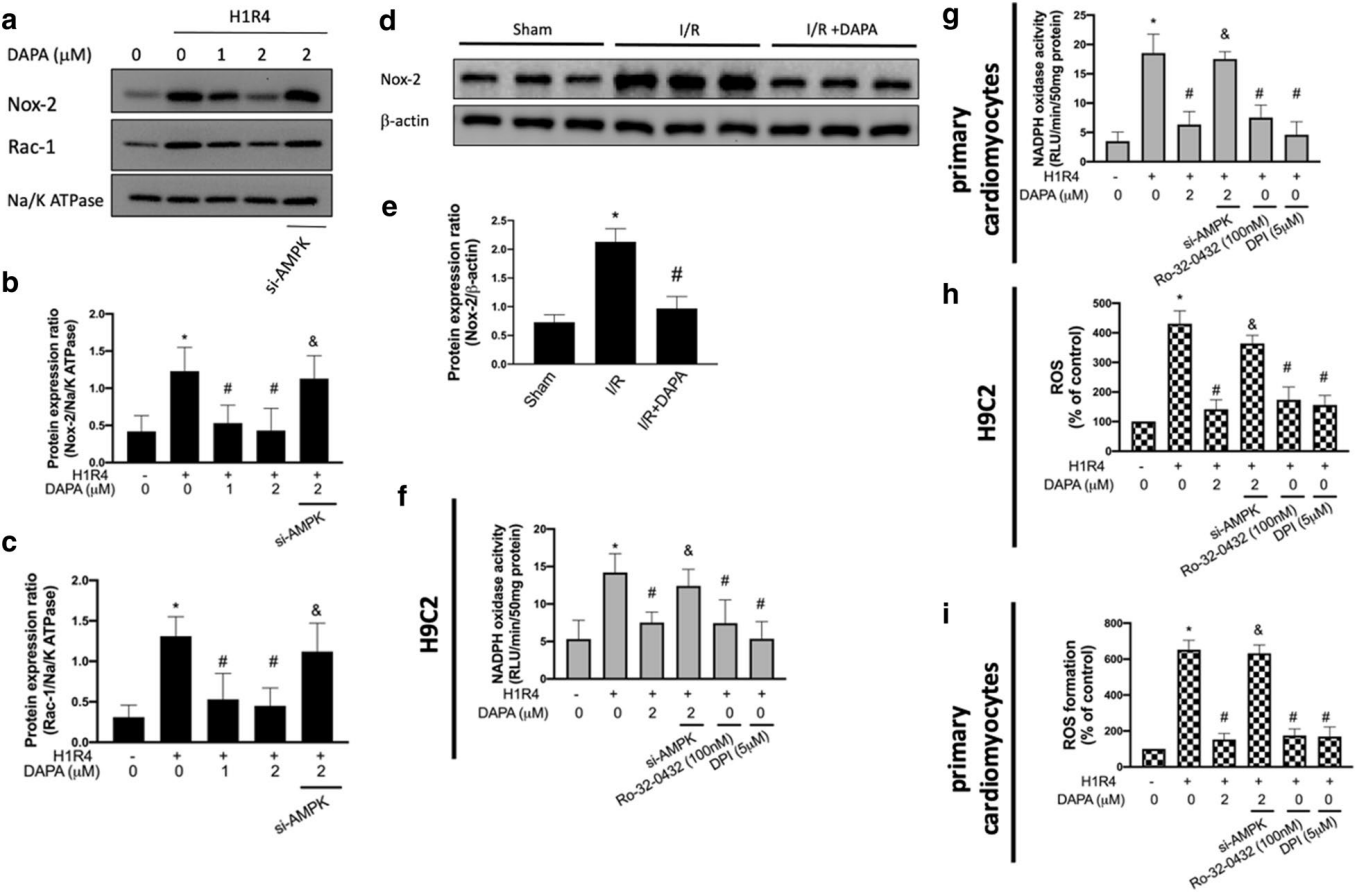
Dapagliflozin (DAPA) prevents the upregulation of ROS via AMPK/ PKC/ NADPH oxidase signaling. Representative Western blot images (**a**) and relative densitometric bar graphs of Nox-2/Na/K ATPase (**b**) and Rac-1/Na/K ATPase (**c**) in H9c2 cells exposed to hypoxia for 1 h and reoxygenation for 4 h (H1R4) were shown. Protein expression levels of Nox-2 and β-actin in ventricular tissue from sham control, ischemia/reperfusion (I/R), and I/R plus DAPA treatment animals (**d**, **e**), eight animals in each group, were examined. Activity of NADPH oxidase in H9c2 cells (**f**) and primary cardiomyocytes (**g**) was measured. ROS generation was measured using a flow cytometry to examine the fluorescent intensity of H9c2 cells (**h**) and primary cardiomyocytes (**i**). For in vitro experiments, the data were presented as the mean ± SD of three biological replicates at three separate times. (* indicating *p* < 0.05 compared with the control group; # indicating *p* < 0.05 compared to H1R4 condition or I/R without DAPA treatment; & indicating *p* < 0.05 compared with the DAPA-treated cells in H1R4 condition)

NADPH oxidase (Nox) is one of the major sources of ROS production after myocardial reperfusion [17]. The main Nox isoform expressed in cardiomyocytes was Nox-2, which required additional protein subunits, such as Rac1, to become fully activated [18]. Previously, it has been indicated that AMPK may inhibit the generation of ROS via suppression of PKC, which in turn hindered the activation of Nox in a rat model of AMI [16]. As such, we examined whether DAPA attenuated oxidative stress in cardiac myoblast H9c2 cells through inhibition of Nox mediated by the AMPK/PKC pathway. As expected, the expression of Nox-2 and Rac-1 in H9c2 cells exposed to H/R injury (Fig. 2a, b, c) and Nox-2 in ventricular tissues with I/R injury (Fig. 2d, e) were all up-regulated. These findings were attenuated by DAPA treatment. In addition, the activity of Nox in H9c2 cells (Fig. 2f) and in primary cardiomyocytes (Fig. 2g), the concentration of ROS in H9c2 cells (Fig. 2h) and in primary cardiomyocytes (Fig. 2i) were all promoted under H/R injury. Nevertheless, the H/R-induced Nox activation and ROS production were abrogated by the treatment of DAPA. Moreover, we showed that silencing of AMPK abolished the effects of DAPA on suppression of Nox-2 and Rac-1 (Fig. 2a, b, c). Similarly, Nox activity (Fig. 2f, g) and ROS generation (Fig. 2h, i) were reversed in cells transfected with si-AMPK. Furthermore, we demonstrated that PKC involved in the H/R-induced Nox activation as administration of Ro-32-0432 (a selective PKC inhibitor) prevented an increase in Nox activity (Fig. 2f, g). Also, we showed that ROS production was mediated by PKC and Nox as treatment of Ro-32-0432 and DPI (a Nox inhibitor) both repressed the H/R-stimulated ROS production (Fig. 2h, i). In concert with previous results, our data suggested that DAPA protected H9c2 cells and cardiomyocytes against oxidative stress following H/R injury through modulation of AMPK/ PKC/ Nox signaling.

### DAPA reverses the hypoxia/reoxygenation-reduced PGC-1α expression and mitochondrial biogenesis through AMPK/ PKC/ NADPH oxidase pathway

**Fig. 3:**
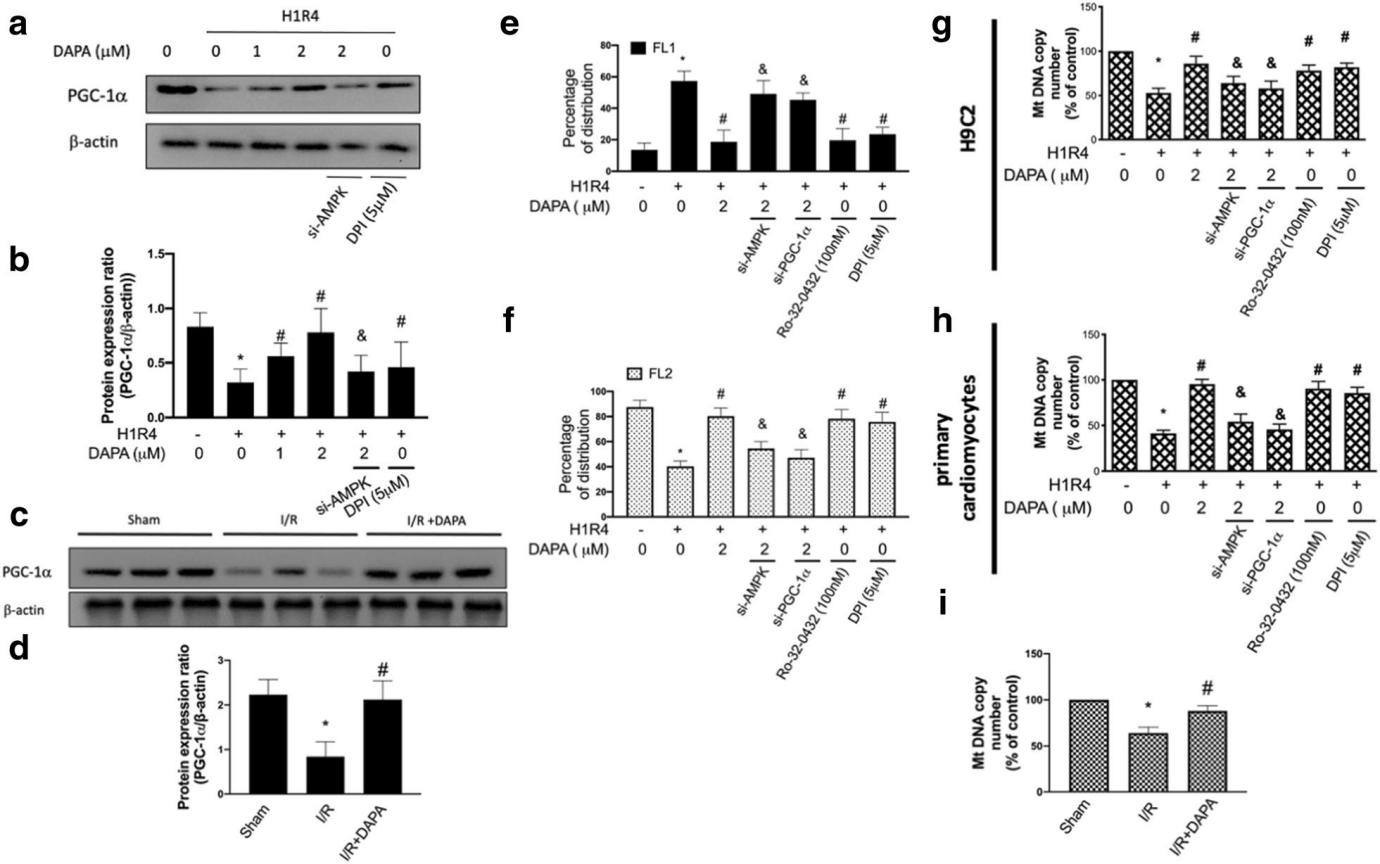
Dapagliflozin (DAPA) prevents hypoxia/reoxygenation-induced PGC-1α downregulation and dysfunction of mitochondrial biogenesis. Representative Western blot images (**a**) and relative densitometric bar graphs (**b**) of PGC-1α/β-actin in H9c2 cells exposed to hypoxia for 1 h and reoxygenation for 4 hr (H1R4) were shown. In some cases, cells were transfected with AMPK siRNA 48 hr or pretreated with DPI before exposure to hypoxia/reoxygenation. Representative Western blot images (**c**) and protein expression levels of PGC-1α and β-actin in ventricular tissue from sham control, ischemia/reperfusion (I/R), and I/R plus DAPA treatment animals (**d**), eight animals in each group, were shown. Percentage of cells expressing JC-1 monomers (green fluorescence; FL1) (**e**) and JC-1 aggregates (red fluorescence; FL2) (**f**) were assessed using flow cytometry. Mitochondrial DNA copy numbers were examined after stimulation of hypoxia/reoxygenation in H9c2 cells (**g**) and primary cardiomyocytes (**h**). Mitochondrial DNA copy numbers in ventricular tissue from sham control, ischemia/reperfusion (I/R), and I/R plus DAPA treatment animals, eight animals in each gruop, were measured (**i**). For in vitro experiments, the data were presented as the mean ± SD of three biological replicates at three separate times. (* indicating *p* < 0.05 compared with the control group; # indicating *p* < 0.05 compared to H1R4 condition or I/R without DAPA treatment; & indicating *p* < 0.05 compared with the DAPA-treated cells in H1R4 condition)

PGC-1α plays a central role in the regulation of the mitochondrial biogenesis and antioxidant gene expression [19] and involves in the AMPK-dependent control of ROS [5]. Here, we showed that the expression of PGC-1α was suppressed following H/R injury whereas treatment of DAPA maintained the amount of PGC-1α. Moreover, the silence of AMPK abrogated this effect (Fig. 3a, b), suggesting that reservation of PGC-1α expression by DAPA was related to AMPK activation. Downregulation of PGC-1α by I/R injury attenuated by DAPA treatment was confirmed by animal study (Fig. 3c, d). Besides, treatment of DPI to inhibit Nox attenuated the downregulation of PGC-1α under H/R injury (Fig. 3a, b), suggesting oxidative stress participated in the downregulation of PGC-1α after H/R injury. Taken together, the downregulation of PGC-1α by H/R or I/R injury could be attenuated via oxidative stress inhibition, and via AMPK activation by DAPA treatment.

Next, the membrane-permeant JC-1 dye was utilized to measure mitochondrial membrane potential and apoptosis by flow cytometry. We observed a higher percentage of cells expressing JC-1 red fluorescence (FL2) in control cells, while cells subjected to H/R injury displayed the downregulated JC-1 red fluorescence (FL2) and the increased green fluorescence (FL1). With the administration of DAPA, cells under H/R condition showed a similar red/green fluorescence intensity ratio as control cells, indicating the protective effect of DAPA on the apoptosis caused by H/R injury. Nevertheless, this protective effect was not observed in H/R-injured cells treated with si-AMPK or si-PGC-1α as there were more apoptotic cells expressing green fluorescence (FL1) in these groups. On the other hand, the percentages of FL2/FL1 in cells co-treated with Ro-32-0432 or DPI were comparable to control cells, indicating that PKC and Nox-2 participated in the induction of apoptosis by H/R (Fig.3e, f). As for mitochondrial DNA copy number, we showed that DAPA limited the H/R-induced dropping of DNA copy number in H9c2 cells. Likewise, knockdown of AMPK and PGC-1α demonstrated a lower mitochondrial DNA copy number (Fig. 3g), indicating upregulation of AMPK and PGC-1α was required for the preservation. Treatment of cells with Ro-32-0432 or DPI showed that repression of PKC and Nox-2 was able to avoid the H/R-reduced mitochondrial DNA copy number. These findings were further confirmed in primary cardiomyocytes (Fig. 3h). In addition, investigation of mitochondrial DNA copy numbers in ventricular tissues also revealed that DAPA treatment protected against I/R-reduced mitochondrial DNA copy numbers in rats (Fig. 3i).

###  DAPA diminishes hypoxia/reoxygenation-induced apoptosis and mitigates the activation of caspase-3 via AMPK/ PKC/ NADPH oxidase/ PGC-1α pathway

**Fig. 4:**
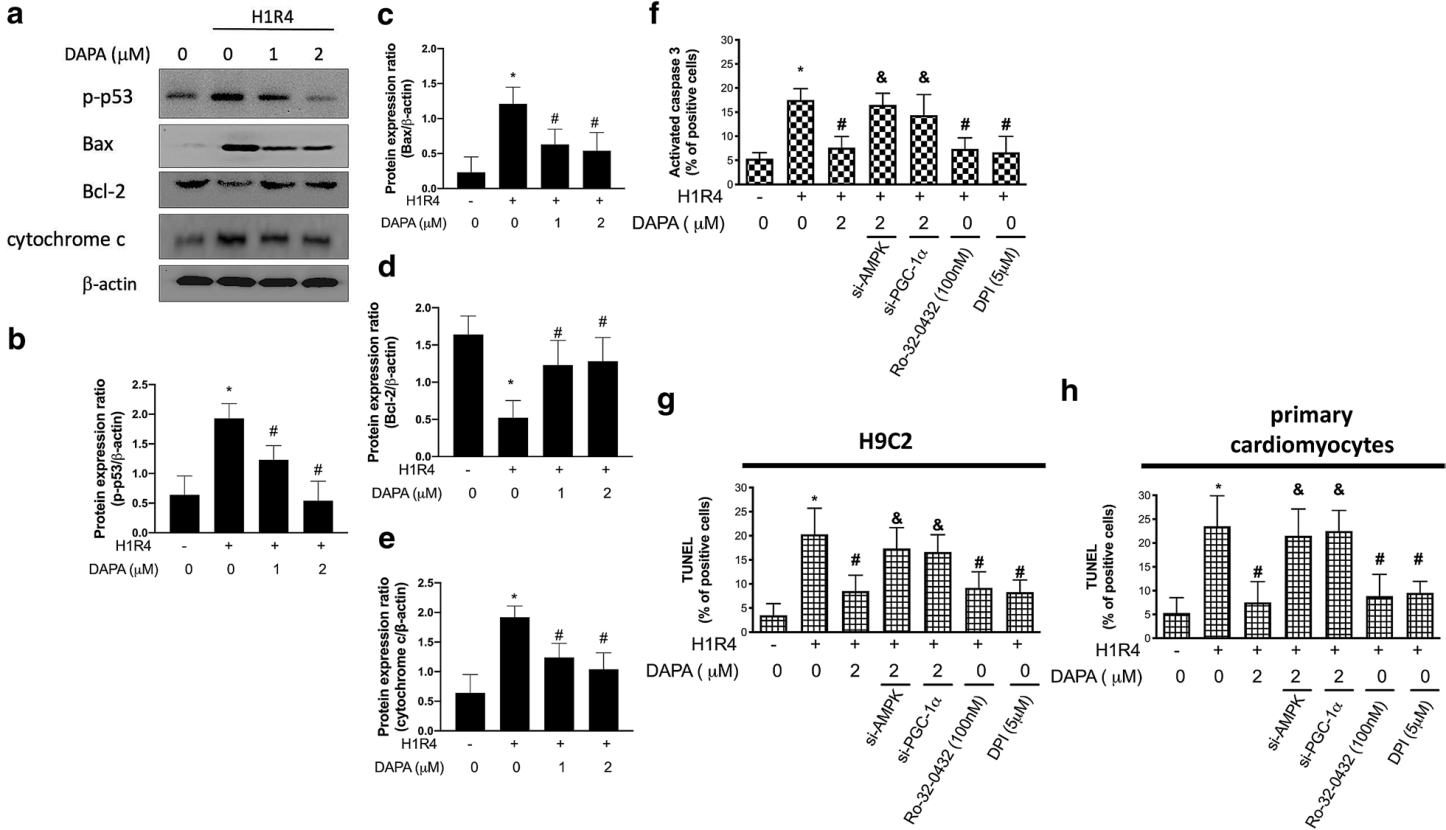
Dapagliflozin (DAPA) prevents hypoxia/reoxygenation-induced apoptosis through AMPK-modulated mitochondrial-dependent pathway. Representative Western blot images (**a**) and relative densitometric bar graphs (**b**–**e**) of mitochondrial-dependent apoptotic markers in H9c2 cells exposed to hypoxia for 1 h and reoxygenation for 4 h (H1R4) were shown. Antiapoptotic effect of DAPA was further confirmed by caspase 3 activity (**f**). TUNEL assay was used for investigating apoptotic cells in H9c2 cells (**g**) and primary cardiomyocytes (**h**). The data were presented as the mean ± SD of three biological replicates at three separate times (* indicating *p* < 0.05 compared with the control group; # indicting *p* < 0.05 compared to H1R4 condition without DAPA treatment; & indicating *p* < 0.05 compared the DAPA-treated cells in H1R4 condition)

Aside from the JC-1 assay, we also examined the expression of various apoptosis-related proteins (Fig. 4a). The expression level of phosphorylated p53 was upregulated following H/R insult (Fig. 4a, b), and treatment of DAPA dose-dependently downregulated it. We also showed a greater expression of Bax and a less expression of Bcl-2 in cells experienced H/R condition, but DAPA administration seemed to reduce apoptotic susceptibility (Fig. 4a, c, d). Besides, the expression of cytochrome *c* in each group corresponded with the abovementioned results and showed that DAPA inhibited the H/R-induced cytochrome *c* elevation (Fig. 4a, e). Results from the caspase 3 assessments demonstrated that DAPA impeded the H/R-stimulated caspase 3 activation (Fig. 4f). Knockdown of AMPK or PGC-1α in DAPA-treated cells exhibited upregulation of caspase 3 (Fig. 4f), indicating that the increase of AMPK or PGC-1α was necessary to the inhibitory effect of DAPA on apoptosis. Application of Ro-32-0432 or DPI showed that downregulation of PKC and Nox-2 repressed the activation of caspase 3 (Fig. 4f). This conclusion was further supported by TUNEL assay in H9c2 cells (Fig. 4g) and in primary cardiomyocytes (Fig. 4h). Taken together, these data demonstrated that DAPA was able to suppress the apoptosis of H9c2 cells in response to H/R insult, which may be mediated by AMPK/ PKC/ Nox/ PGC-1α signaling.

### DAPA protects the heart from the damage caused by ischemia/reperfusion injury

**Fig. 5:**
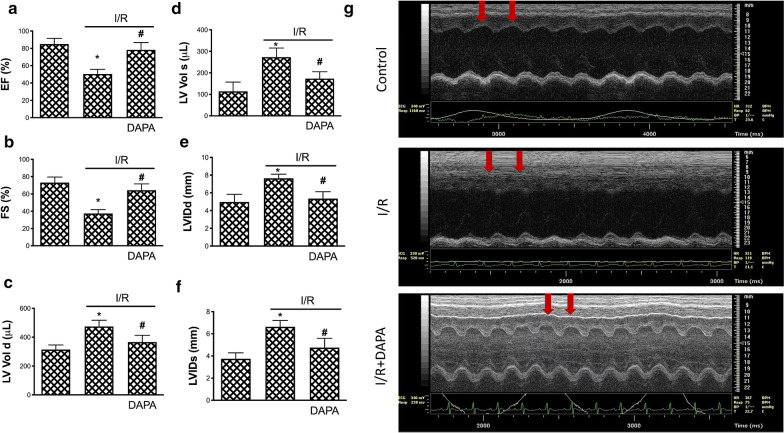
Dapagliflozin (DAPA) protects heart function on ischemia/reperfusion injury. Measurement in echocardiography, including (**a**) ejection fraction (EF), (**b**) fractional shortening (FS), (**c**) left ventricular end-diastolic volume (LV Vol d), (**d**) left ventricular end-systolic volume (LV Vol s), (**e**) left ventricular internal dimension at end-diastole (LVIDd), and (**f**) left ventricular internal dimension at end-systole (LVIDs), were shown. (**g**) Representative echocardiographic M-mode images from animals in different conditions revealed reduced motion of anterior wall of left ventricle caused by ischemia/reperfusion (I/R) (middle panel), which was attenuated by DAPA treatment (lower panel). Red arrows indicate the motion of anterior wall of left ventricle. The data were presented as the mean ± SD in sham control, ischemia/reperfusion (I/R), and I/R plus DAPA treatment animals, eight animals in each group (* indicating *p* < 0.05 compared with the control group; # indicating *p* < 0.05 compared to I/R without DAPA treatment)

We next studied the effect of DAPA on the cardiac function of the AMI rats, which were subjected to 1 h LAD occlusion followed by reperfusion for 4 days, representing I/R injuries. Data from echocardiography showed that the ejection fraction and fractional shortening were significantly decreased in the heart of I/R injured rats, while administration of DAPA prevented these two values from dropping (Fig. 5a, b). In addition, the increase in left ventricular end-diastolic and end-systolic volume as well as the left ventricular internal dimension at end-diastole and end-systole were reduced in the I/R injured rats with DAPA treatment compared to those I/R injured rats without DAPA treatment (Fig. 5c, d, e, f). Representative echocardiographic M-mode images were presented (Fig. 5g). These data suggested that DAPA treatment reduced the dilatation and contractile dysfunction of left ventricle caused by I/R injury.

**Fig. 6:**
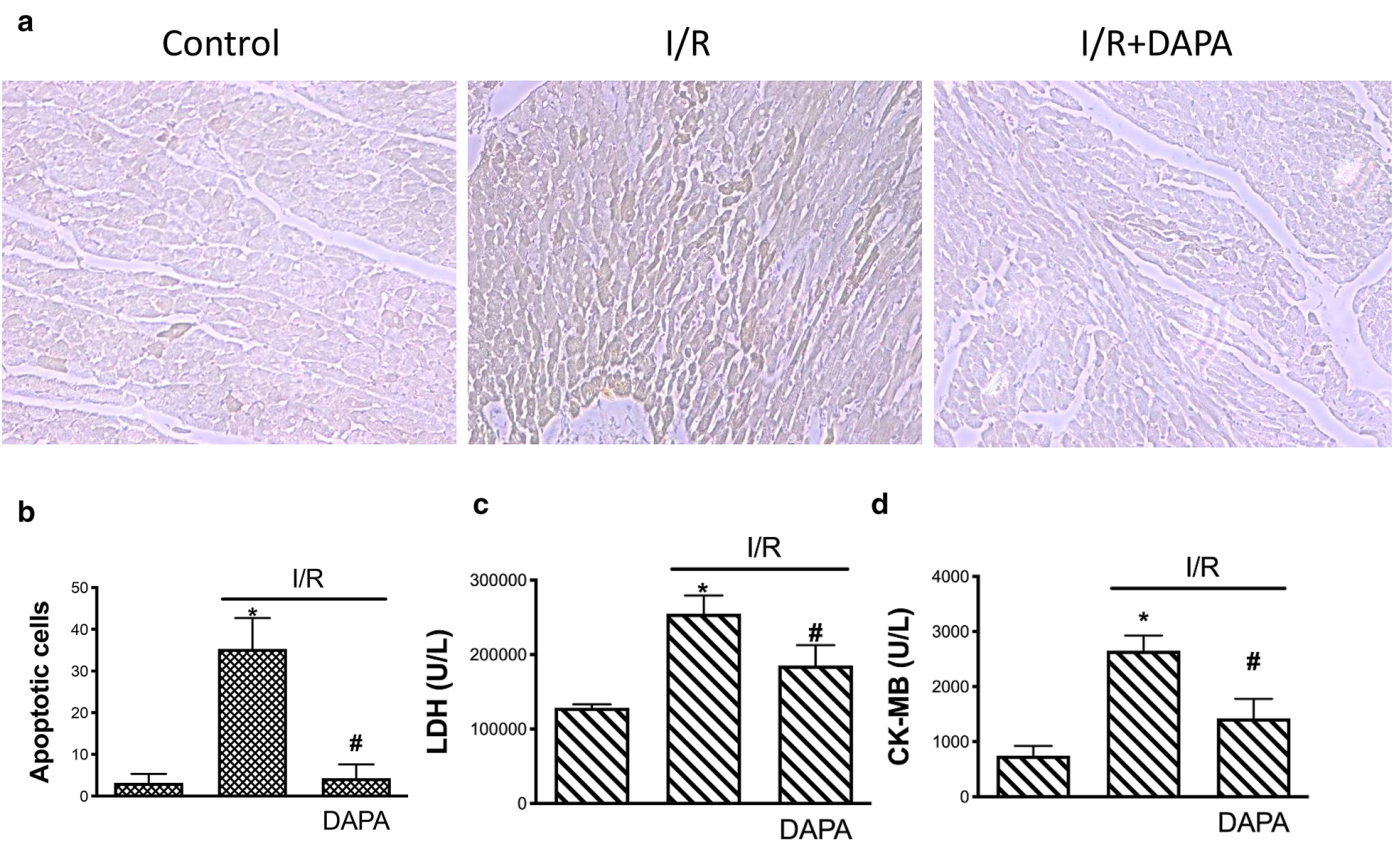
Anti-apoptotic effect of dapagliflozin (DAPA) on ischemia/reperfusion injured animals. Representative images of TUNEL staining of the cardiac tissues (**a**) and quantification of the apoptotic areas (**b**) were shown. The plasma concentration of myocardial damage markers, lactate dehydrogenase (LDH) (**c**) and creatine kinase-MB (CK-MB) (**d**), in control animals and ischemia/reperfusion (I/R) animals with or without DAPA treatment were checked. The data were presented as the mean ± SD of eight animals in each group. (* indicating *p* < 0.05 compared with the control group; # indicating *p* < 0.05 compared to I/R without DAPA treatment)

In order to investigate the effect of DAPA on the reduction of apoptosis in vivo, we utilized the TUNEL assay to evaluate the apoptosis in the heart of AMI rats. The percentage of TUNEL-positive cells was greater in the I/R injury group compared to the sham group (Fig. 6a, left and middle panel) and DAPA treatment significantly reduced the TUNEL-positive cells in I/R injury group (Fig. 6a, middle and right panel), indicating the administration of DAPA ameliorated the I/R-elicited apoptosis in the heart. Moreover, we showed that the concentration of two enzymes that were used to predict and estimate myocardial injury and infarct size, LDH and CK-MB, were both elevated in the blood following I/R injury (Fig. 6c, d). Nevertheless, these changes were attenuated in the rats receiving DAPA treatment, which was in agreement with the finding of less myocardial apoptosis. Taken together, these results confirmed that the administration of DAPA ameliorated the myocardial apoptosis and damage following I/R stimulus.

## Discussion

**Fig. 7:**
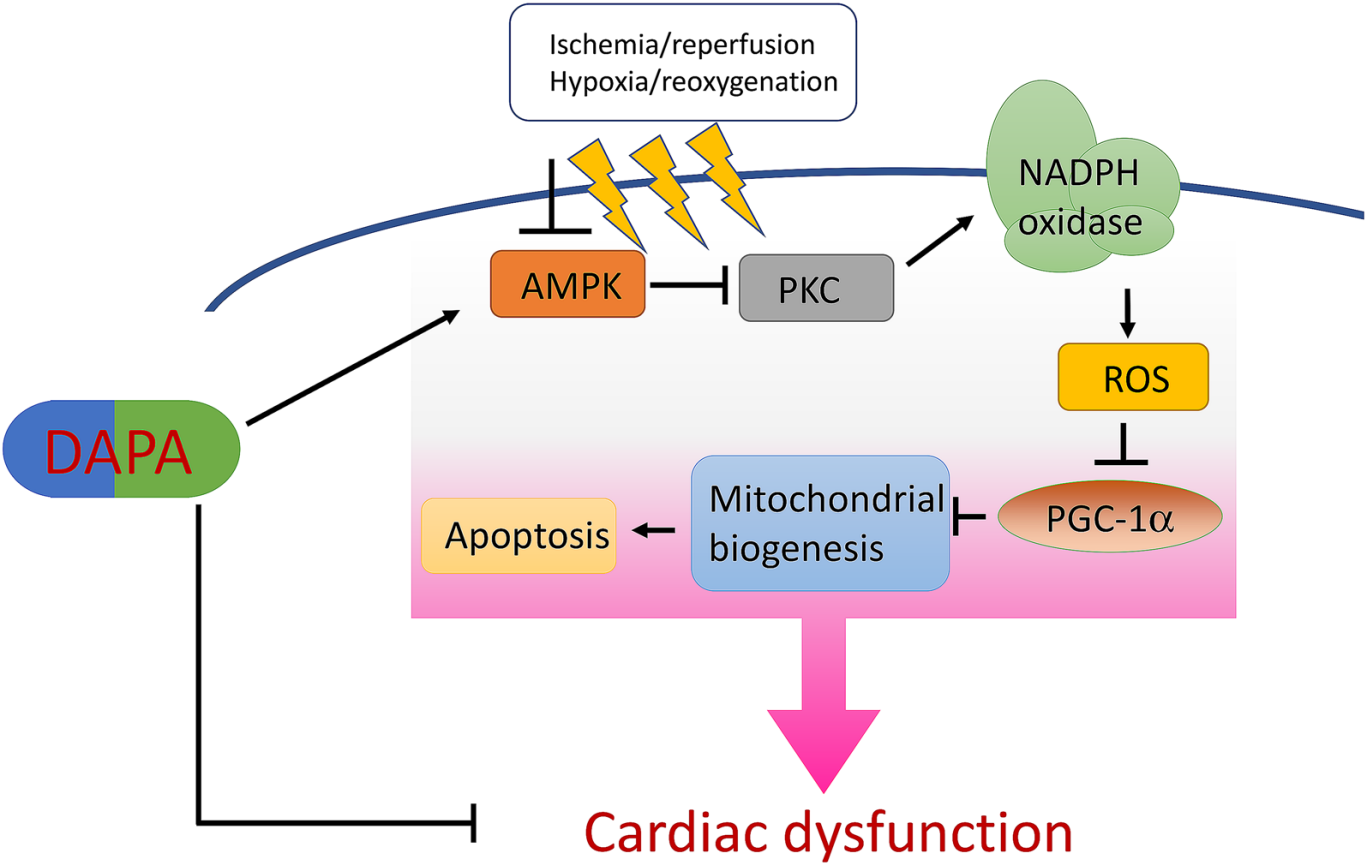
Schematic diagram showing protective signaling of dapagliflozin (DAPA) on ischemia/reperfusion-caused cardiac dysfunction

Over the past few years, mounting evidence suggested that SGLT2 inhibitors, which were first used as antidiabetic medications, exerted preferentially favorable effects to reduce heart failure and cardiovascular death in DM patients [20, 21]. Among these SGLT2 inhibitors, the benefits of DAPA have been demonstrated in various clinical studies [9, 11, 12, 22]. One of the recent in vivo experiments even indicated that acute DAPA administration possessed the cardioprotective effects in rats subjected to I/R injury [23]. Several pieces of research have unraveled how DAPA contributes to cardiovascular benefits. First, DAPA may be able to regulate the ionic homeostasis in cardiomyocytes. For instance, DAPA was found to lower the amplitude of the calcium transients in control and type I DM ventricular myocytes [24]. Also, it inhibited the activity of Na^+^/H^+^ exchanger (NHE) and downregulated cytosolic Na^+^ [25], and blockage of NHE has been revealed to reduce ischemic Na^+^ overload and enhance the post-ischemic contractile recovery in the heart of rats with I/R injury [26]. Secondly, DAPA reduced the oxidative stress and prolonged ventricular repolarization via augmentation of mitochondrial function. It has been shown to preserve the depolarized mitochondrial membrane potential, and enhance cytosolic calcium homeostasis [27]. In line with these findings, we showed that the administration of DAPA suppressed the production of ROS. Besides, it reversed the altered mitochondrial membrane potential and DNA copy number following H/R insult, which attenuated the subsequent apoptosis. Most importantly, our data further elucidated that AMPK activation played a significant role in the DAPA-regulated mitochondrial energetics (Fig. 7).

As an energy sensor, AMPK orchestrated multiple metabolic responses to energy deprivation, and modulation of AMPK has been thought to be the key to the prevention of reinfarction or apoptosis. Certainly, numerous clinically approved medications that upregulated AMPK, such as metformin [28] and aspirin [29, 30], have been shown to hold the potential to ameliorate myocardial I/R injury. Here, we provided the evidence to substantiate that AMPK phosphorylation by DAPA activated PGC-1α, the crucial modulator of mitochondrial function and oxidative metabolism in the heart. Previously, AMPK has been shown to directly phosphorylate PGC-1α at threonine-177 and serine-538 in the skeletal muscle [31], and our results suggested that the DAPA-induced AMPK upregulated PGC-1α via PKC/Nox pathway. PGC-1α deficiency has been proven to diminish normal cardiac function [32], and it has been shown that AMPK/PGC-1α axis participated in the acetylcholine-induced [33] and metformin-associated [34] cardioprotective effects. In the present study, we showed that the restoration of PGC-1α by DAPA reduced the mitochondrial dysfunction and myocardial apoptosis following H/R insult, which was consistent with various studies showing PGC-1α mediated mitochondrial oxidative damage and apoptotic susceptibility [35, 36].

Besides, AMPK activation has been known to suppress oxidative stress via multiple mechanisms. For instance, AMPK activation eliminated the hyperglycemia-induced mitochondrial ROS through elevation of manganese superoxide dismutase and enhancement of mitochondrial biogenesis in human umbilical vein endothelial cells (HUVECs) [37]. Additionally, AMPK phosphorylation increased the expression of mitochondrial uncoupling protein-2, leading to the inhibition of both superoxide anions and prostacyclin synthase nitration in HUVECs [38]. AMPK was also implicated in the regulation of Nox pathway, which was indispensable for the accumulation of oxidative stress following myocardial I/R injury [39]. Various studies have suggested that AMPK activation mitigated glucose-induced oxidative stress through Nox suppression [40, 41]. Loss of AMPK activity has been reported to elevate the expression of several Nox subunits and the oxidase-mediated superoxide production in endothelial cells [42]. Moreover, it has been demonstrated that AMPK phosphorylation by rosiglitazone may inhibit PKC, which impeded the Nox activation and glucose-induced oxidative stress in HUVECs [40]. Another study showed a similar finding that AMPK may hamper the PKC-mediated accumulation of ROS by suppression of Nox [16]. Likewise, we showed that the upregulation of AMPK was associated with the repression of PKC, leading to a diminution in Nox-2 activity in cells treated with DAPA. Among various Nox isoforms, Nox-1 and Nox-2 have been demonstrated to mediate myocardial oxidative damage during reperfusion, and Nox-2 also participated in the generation of oxidative stress post-reperfusion [39]. Our results disclosed that administration of DAPA reduced the Nox-2-mediated oxidative stress. Further investigation of whether DAPA attenuates organ damage and displays Nox-2 suppressive effect in other I/R injury models may represent an intriguing subject.

Apart from the in vitro experiment, we examined the effects of DAPA on cardiac function and myocardial apoptosis. Data from echocardiography revealed that DAPA administration improved the measurement of left ventricular function affected by I/R injury, and this was in line with a recent study revealing the positive effect of DAPA on the enhancement of left ventricular function of type II DM patients with stable heart failure [22]. Moreover, we showed that treatment of DAPA attenuated the rise in the levels of two AMI-associated enzymes (CK-MB and LDH) in response to I/R injury, which was in conformity with a study showing SGLT2 inhibitor exhibited cardioprotective effects and downregulated the serum CK-MB and LDH in DM rats [43]. Taken together, DAPA may have a protective effect on cardiac function in the setting of AMI.

## Conclusions

This current study demonstrated that DAPA reduced the extent of myocardial injury and apoptosis and improved cardiac contractile function in the I/R injured heart. Our data revealed that AMPK/ PKC/ Nox/ PGC-1α signaling mediated the cardioprotection of DAPA against I/R injury and provided molecular insight into the probable benefit of DAPA for patients with AMI.

## Data Availability

The datasets generated and analyzed during the current study are available from the corresponding author on reasonable request.

## Abbreviations

DAPA: Dapagliflozin
I/R: Ischemia/reperfusion
AMPK: Adenosine 5’-monophosphate activated protein kinase
PKC: Phosphorylated protein kinase C
H/R: Hypoxia/reoxygenation
PGC-1α: Proliferator-activated receptor-gamma coactivator 1-alpha
ATP: Adenosine triphosphate
ROS: Reactive oxygen species
SGLT2: Sodium-glucose cotransporter 2
DM: Diabetes mellitus
DMEM: Dulbecco’s modified Eagle’s medium
FBS: Fetal bovine serum
EDTA: Ethylenediaminetetraacetic acid
JC-1: 5,58,6,68-tetraethylbenzimidazolcarbocyanine iodide
DPI: Diphenyleneiodonium chloride
DHE: Dihydroethidium
