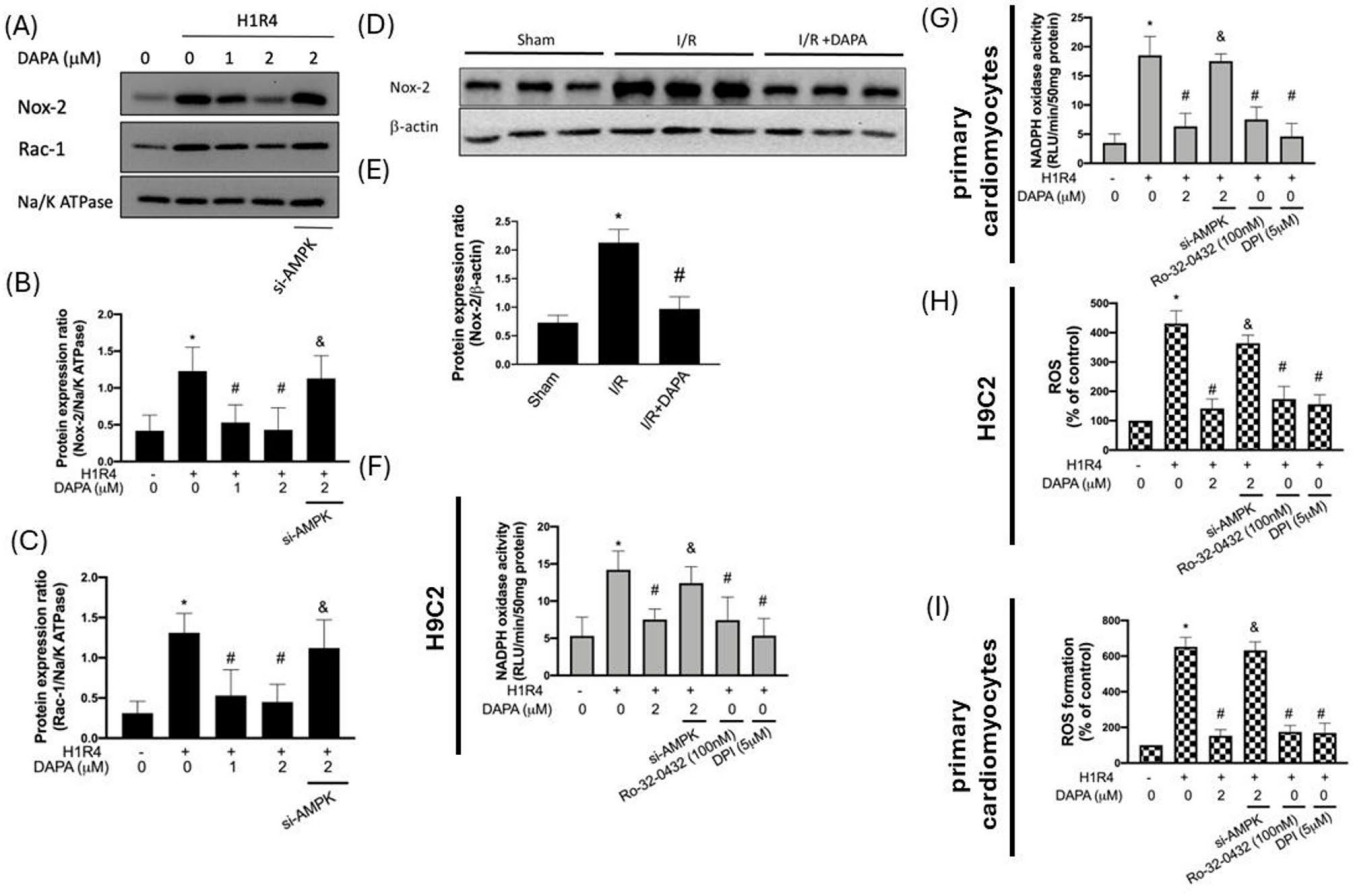# Correction: Dapagliflozin attenuates hypoxia/reoxygenation-caused cardiac dysfunction and oxidative damage through modulation of AMPK

**DOI:** 10.1186/s13578-026-01606-y

**Published:** 2026-06-19

**Authors:** Kun‑Ling Tsai, Pei‑Ling Hsieh, Wan‑Ching Chou, Hui‑Ching Cheng, Yu‑Ting Huang, Shih-Hung Chan

**Affiliations:** 1 Department of Physical Therapy, College of Medicine, National ChengKung University, Tainan, Taiwan; 2 Institute of Allied Health Sciences, Collegeof Medicine, National Cheng Kung University, Tainan, Taiwan; 3 Departmentof Anatomy, School of Medicine, China Medical University, Taichung, Taiwan; 4 Department of Internal Medicine, College of Medicine, National Cheng KungUniversity Hospital, National Cheng Kung University, Tainan, Taiwan

**Correction: Cell Biosci (2021) 11:44**

 10.1186/s13578-021-00547-y

In this article [1], the authors claim that Fig. 2, which reports Western blot data, was not matched to the original blot, which was erroneous. The corrected version is reported here. The authors claim that this correction does not affect the study’s conclusion. The authors report that the β-actin of Fig. 2D was found to be mismatched in the original publication. The incorrect and correct versions of Fig. 2 are displayed below.

The original article has been corrected.

Incorrect Fig. 2:


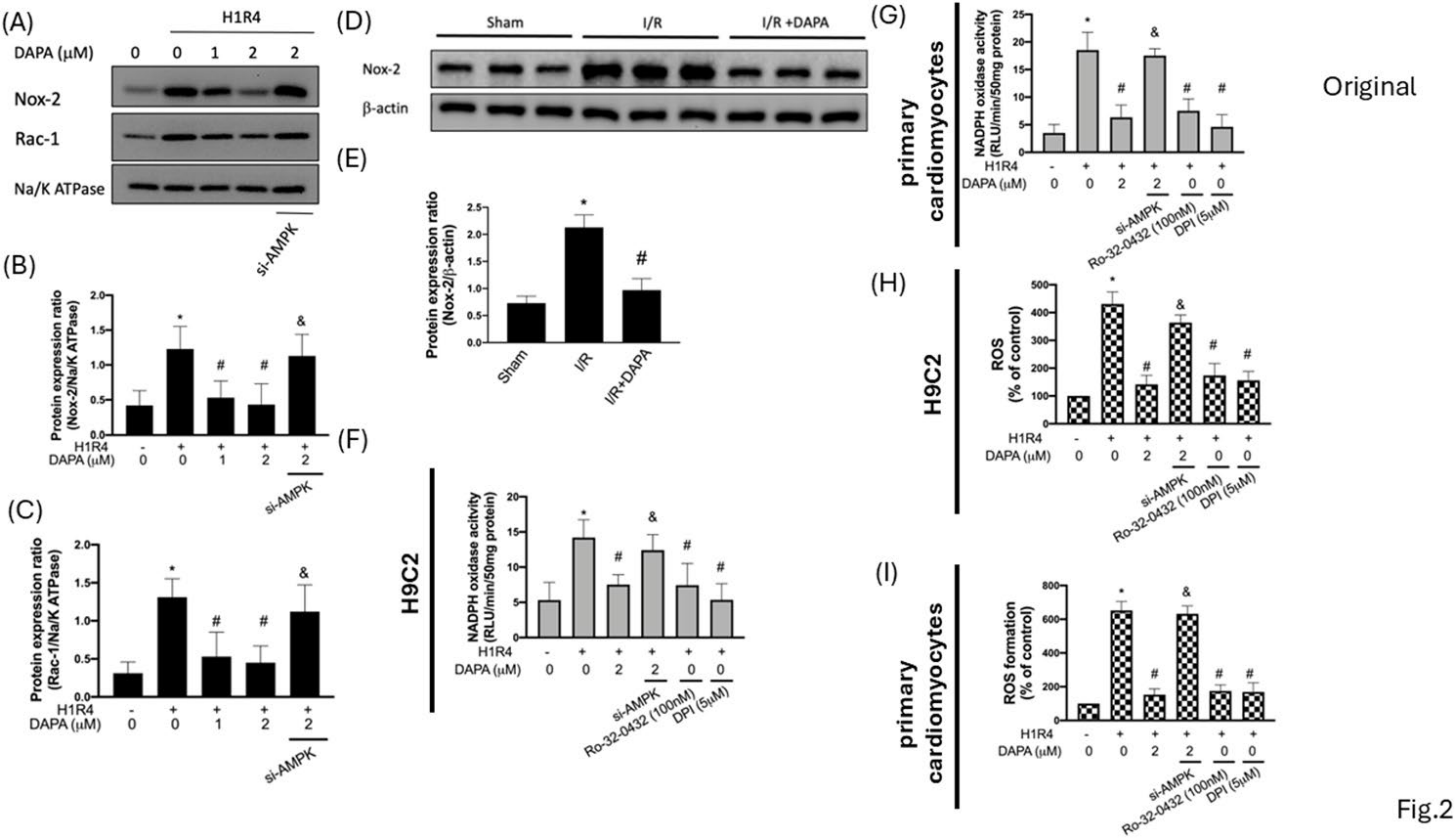


Correct Fig. 2: